# Evaluation of Powder Metallurgy Workpiece Prepared by Equal Channel Angular Rolling

**DOI:** 10.3390/ma16020601

**Published:** 2023-01-08

**Authors:** Róbert Kočiško, Tibor Kvačkaj, Jana Bidulská, Róbert Bidulský, Patrik Petroušek, Imrich Pokorný, Miloslav Lupták, Marco Actis Grande

**Affiliations:** 1 Department of Plastic Deformation and Simulation Processes, Institute of Materials and Quality Engineering, Faculty of Materials, Metallurgy and Recycling, Technical University of Kosice, Park Komenského 11, 04001 Kosice, Slovakia; 2 Bodva Industry and Innovation Cluster, Budulov 174, 04501 Moldava nad Bodvou, Slovakia; 3 Department of Applied Science and Technology (DISAT), Politecnico di Torino, Viale T. Michel 5, 15121 Alessandria, Italy

**Keywords:** powder metallurgy, equal channel angular rolling, formability, diametrical compression test, finite element method, stress triaxiality

## Abstract

The aim of the article is to examine the workability of sintered powder material of aluminum alloy (Alumix 321) through severe plastic deformations under the conditions of the equal channel angular rolling (ECAR) process. Accordingly, the stress–strain analysis of the ECAR was carried out through a computer simulation using the finite element method (FEM) by Deform 3D software. Additionally, the formability of the ALUMIX 321 was investigated using the diametrical compression (DC) test, which was measured and analyzed by digital image correlation and finite element simulation. The relationship between failure mode and stress state in the ECAR process and the DC test was quantified using stress triaxiality and Lode angle parameter. It is concluded that the sintered powder material during the ECAR processing failure by a shearing fracture because in the fracture location the stress conditions were close to the pure shear (*η* and $\bar{\theta }$ ≈ 0). Moreover, the DC test revealed the potential role as the method of calibration of the fracture locus for stress conditions between the pure shear and the axial symmetry compression.

## 1. Introduction

Aluminum alloys prepared by powder metallurgy are still the subject of intensive development for the improvement of utility properties, which require their demanding structural application. The technological procedure of powder metallurgy brings porosity into the material, which mainly adversely affects the mechanical properties. Therefore, it is necessary to deal with the development of new procedures for the synthesis of powder materials that ensure full density and strength at low processing temperatures. Currently, several innovative techniques based on severe plastic deformations (SPD) are available, which effectively improve mechanical properties. Several SPD methods have been applied to the synthesis of powder materials such as Equal Channel Angular Pressing (ECAP) [1,2,3,4], Equal Channel Angular Pressing-Back Pressure (ECAP-BP) [5,6], High pressure torsion (HTP) [7,8], Accumulative Roll Bonding (ARB) [9], etc. The severe shear deformation in these processes ensures better contact between the particles and improves the break of the oxide layers on the particles. The mentioned techniques produce relatively small samples. Some novel SPD continuous shear deformation processes such as Conshearing techniques, continuous confined strip shearing (C2S2) [10,11,12], ECAP-Conform (ECAP-C) [13,14], or equal channel angular rolling (ECAR) [15,16,17,18,19,20] based on the ECAP method appear to be suitable for continuous sintering of the powder metallurgy (PM) materials. It should be noted that methods such as C2S2, ECAP-C, and ECAR seem to be the same, but each of them has its own specific technical solution and, thus, performance in generating ultrafine grained (UFG) structures. The ECAR process is a method that involves large shear plastic deformation in a billet by moving through a die containing two intersecting channels of identical cross-sections. The input channel is created by groove roll, which ensures the movement of the sample through the main deformation zone. The die angular Φ between channels can vary in the range from 90° to 135°. The process can be repeated by extruding the sample through the same matrix.

The ECAR process in the PM area has not been widely examined and published yet [3,18]. It is important to note that bulk forming of the PM materials is needed to realize without failure or damage observation. Therefore, limitations are given by the appearance of a surface or internal cracks within regions that are highly strained, mainly due to the extensive material flow involved by SPD.

Workability commonly refers to the ability of the material to withstand prior to failure in bulk-forming processes. Workability depends on process parameters such as stress, strain, strain rate, friction, forming temperature, and strain hardening as well as on the typical PM features such as porosity. For the assessment of critical workability, values have been proposed various criteria such as Cockroft-Latham (CL), Brozzo, Oyane, Johnson–Cook [21,22,23,24], etc. Cockcroft and Latham fracture criterion, which is based on a critical value of the tensile strain energy per unit of volume, is often used for engineering applications [25]. It was developed for bulk-forming operations and, therefore, applies only to the range of small and negative stress triaxiality [26]. The normalized version of this criterion can be written as a damage factor defined as a normalized criterion (nCL) [27]:

(1) $$C=\int_{0}^{\bar{\varepsilon_{ef}}}\frac{\sigma_{1}}{\bar{\sigma }}.d\bar{\varepsilon }$$

where ***C*** is the calibration constant,$\;\sigma_{1}$ is the maximum tensile principal stress, $\bar{\sigma }$ is the effective stress according to the von Mises, is the effective stress, $\bar{\varepsilon_{ef}}$ is the effective strain at the fracture, and $\bar{\varepsilon }$ is the increment of effective strain. 

The critical failure values necessary for the calculation of the ductile fraction criterion are determined by laboratory tests that use different methods of mechanical stress on the test sample such as tension, pressure, bending, or torsion. It is determined at the moment of occurrence of a violation of the cohesion of the tested sample. In the field of computer simulations of plastic deformation processes, the damage factor is of great importance, because it can reveal a violation of the material in the processing process. 

The method of the diametral compression (DC) test, also called the Brazilian disc test, is usually used to determine the tensile strength of brittle and low-strength materials such as concrete [28], rock material [29], cemented carbides and ceramics [30], pharmaceutical materials in dosage forms [31], and other brittle materials or material with limited plasticity. It was also used for testing materials based on metal powders [32,33,34,35]. The principle of the DC test consists of compressing the cylindrical sample in the radial direction which along the horizontal diameter of the disc will create high normal tensile stresses which will cause a failure of the sample. If the material is homogeneous and isotropic, the normal tensile strength can be derived from the following equation [36]:

(2) $$\sigma_{t}=\frac{2F}{\pi D.t}$$

where F is the value of the applied load and D and t are, respectively, the diameter and the thickness of the specimen.

Despite the fact that the damage factor is a sufficient parameter to reveal the location of a probable material failure, it does not provide information about the mechanism by which the fracture occurred or the state of tension that caused it. The effect of stress state on material fracture strain is possible quantify by using stress triaxiality and Lode angle parameter [37,38,39]. For isotropic materials, the material models can be formulated in terms of three stress invariants (p, q, r) which are defined as:

(3) $$p=-\sigma_{m}=-\frac{1}{3}tr\left(\sigma \right)=-\frac{1}{3}\left(\sigma_{1}+\sigma_{2}+\sigma_{3}\right)$$

(4) $$q=\bar{\sigma }=\sqrt{\frac{3}{2}S:S}=\sqrt{\frac{1}{2}\left[{\left(\sigma_{1}-\sigma_{2}\right)}^{2}+{\left(\sigma_{2}-\sigma_{3}\right)}^{2}+{\left(\sigma_{3}-\sigma_{1}\right)}^{2}\right]}=\sqrt{3J_{2}}$$

(5) r=92detS13=272σ1−σmσ2−σmσ3−σm13=272J313

where σ is the stress tensor, $\sigma_{m}$ is the hydrostatic stress, S is the deviatoric stress tensor, whereas $J_{2}$ and $J_{3}$ are the second and third deviatoric stress invariants, $\bar{\sigma }\;$is Von Mises equivalent stress, the parameter p is positive in compression, but $\sigma_{m}$ is positive in tension.

Stress triaxiality is an important parameter in fracture mechanics and often be used to predict the type of fracture (i.e., ductile or brittle), which is defined as the ratio of hydrostatic stress to Von Mises equivalent stress in Equation (6):

(6) $$\eta =\frac{-p}{q}=\frac{\sigma_{m}}{\bar{\sigma }}$$

For condition biaxial (plane) stress states, the values of the triaxiality factor must always remain in the range $\eta \in \langle -\frac{2}{3};\;\frac{2}{3}\rangle$, while for the general case of three-dimensional multiaxial states, the triaxiality factor can take any value from the range $\eta \in \left(-\infty ;\infty \right)$ [40]. The stress triaxiality is not sufficient to completely characterize the stress state because it does not distinguish between the different shear stress states. This condition is completed by the Lode angle parameter which is defined through the third deviatoric stress *ξ* in Equation (7):

(7) $$\xi ={\left[\;\frac{r}{q}\;\right]}^{3}=cos\left(3\theta \right)=\frac{27}{2}\frac{J_{3}}{q^{3}}$$

The Lode angle parameter in normalize state is expressed as below [41]:

(8) $$\bar{\theta }=1-\frac{6\theta }{\pi }=1-\frac{2}{\pi }arccos\xi$$

The range of the Lode angle is $\bar{\theta }\in \langle 0;\;\pi /3\rangle \;$ and the range of ξ∈〈−1; 1〉 .

This paper deals with the laboratory processing of sintered powder alloy Alumix 321 by the ECAR technology to improve its mechanical properties. Due to the failure of material cohesion during processing, the ECAR process was analyzed in detail using finite element simulations. The workability of the alloy was evaluated using a diametral compression test from which the Damage factor according to nCL was determined. In this work, digital image correlation and finite element simulations were used to characterize the stress state of the DC test specimen. Finally, the effects of stress triaxiality and the Lode angle parameter on the failure mode were analyzed for both the ECAR process and the DC test.

## 2. Materials and Methods

### 2.1. Materials

The material used for the experiment was an aluminum alloy Alumix 321 (Al-Mg-0.95 wt.%; Si-0.49 wt.%; Cu-0.21 wt.%; Fe-0.07 wt.%; lub-1.5 wt.%) supplied by ECKA Granules Austria GesmbH, Ranshofen, Switzerland. This material has a chemical composition very similar to grade aluminum alloy EN AW 6082. The powder was consolidated by pressure 600 MPa to billet with dimensions 10 mm× 10 mm× 55 mm. The heat treatment of the compact billet was carried out in a vacuum furnace at 585 °C for 30 min. By mechanical machining, the billets were adjusted to the size of 7 mm× 7 mm× 55 mm. Five pieces of the billet were used in the laboratory ECAR process. The fracture surfaces were analyzed using a scanning electron microscope Jeol JSM 7000F [42].

### 2.2. The ECAR Processing

**Figure 1:**
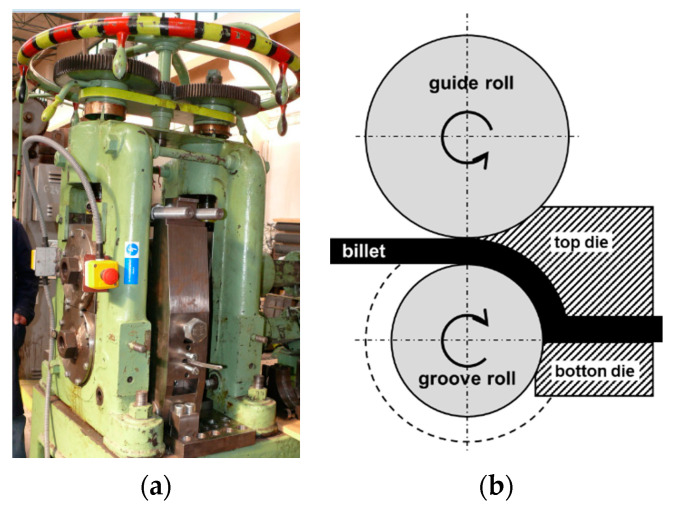
(**a**) The ECAR equipment; (**b**) The schema of the ECAR process.

The ECAR equipment is presented in Figure 1. The groove roller and the guide roller are rotating with constant angular velocity $\omega_{roll}=0.02\;rad/s$ while the other components are stationary. A billet is continuously fed into the groove where the guide roll pressed him into the groove roll which carries away to the intersection of the channels. The output speed of the billet is 2 mm/sec. Both rolls (groove and guide) have a diameter of 210 mm. The walls of the groove rolls are roughened by electro-spark. The high friction force on the wall of the groove and stress from the top die will cause the billet to be passes through the main deformation zone. The laboratory ECAR process of one pass was carried out on the rolling mill DUO at room temperature.

### 2.3. Formability of Alumix 321

For the assessment of critical workability values of the material, Alumix 321 has been used in the DC test which was carried out on a cylinder sample with a diameter of 9 mm and a thickness of 4.3 mm. The Tinius Olsen video-extensometer equipment was used for recording the course of deformation during the DC test. To evaluate the strain, the surface of the specimen was covered with black and white paints which created the stochastic pattern. For displacement measurement and strain monitoring of the specimens, a digital image correlation system was used which was realized in GOM Correlate software (GOM Correlate 2020 version Hotfix 5). The DC test was performed as three-dimensional modes using the commercial metal-forming finite element code by DEFORM-3DTM according to the laboratory test conditions. The nCL damage factor in the point of damage of the sample was determined from the stress–strain analyses of the FEM simulation.

### 2.4. Initial Conditions for Numerical Simulations of the ECAR Process

The ECAR simulation was performed as three-dimensional modes using the software DEFORM-3D^TM^(version v13.0.1 ). The one ECAR pass was simulated through vertical and horizontal channels which form an angle of 90° to each other. The principle of ECAR processing with individual parts is shown in Figure 1b. The initial dimension of the billet was 7 mm in thickness, 7 mm in width, and 250 mm in length.

**Figure 2:**
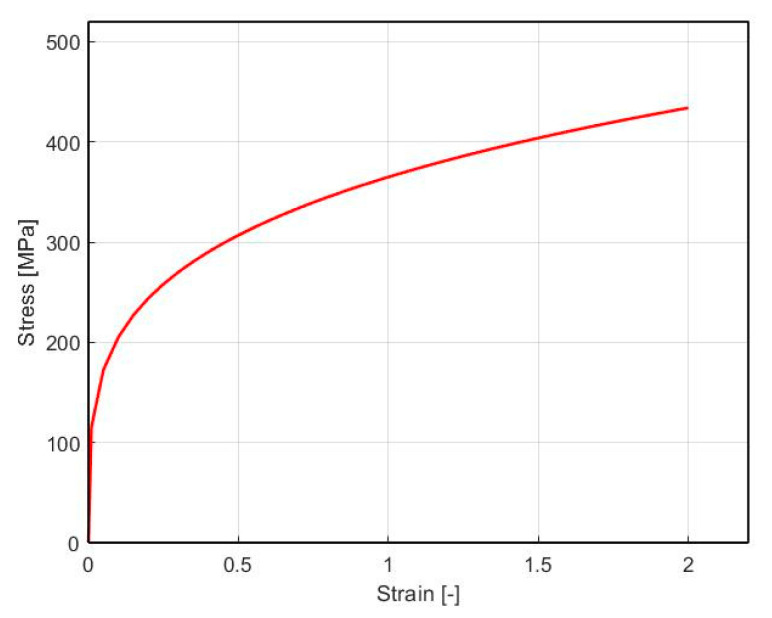
The flow stress data of aluminum alloy.

In the simulations, the billet was defined as a porous material with a bulk density of 90%. For the needs of this research, a simplified material behavior model was used, which is modeled similarly to plastic objects, but the model includes the compressibility of the material. The material was defined as homothetic matrix of a rigid-viscoplastic isotropic and homogeneous material with von Mises model. For the porous material with a rigid visco-plastic von Mises type matrix, many analytical macroscopic yield criteria [43,44] which take into consideration the effect of porosity have been derived by different methods. It should be noted that no stress-based variational model of the macroscopic yield criterion for ductile porous materials is proposed or applied within this study. The flow stress data of the material Alumix 321 for the cold forming process was acquired from the upsetting of the cylindrical test. The test was carried out at a strain rate of $1\;\;s^{-1}$. The homogeneity of plastic strain in the cylinder test sample during pressing was secured with low friction conditions on the contact surface use of a graphite slice. Figure 2 displayed the strain hardening curve as true stress vs. true strain. The relationship among them was fitted according to the Hollomon relationship $\sigma =K.\varphi^{n}$, where K is the effective Von-Misses stress (K=365 MPa), φ—strain, and n is the strain hardening exponent (n=0.25). The maximal principal stress at which the material went into the plastic state at the uniaxial state of stress has a value of $\sigma_{1}=115\;MPa$, while the maximum shear stress according to the Von Mises condition is$\;\tau_{max}=66\;MPa$.

The tools of the ECAR equipment (the die and working rolls) were considered a rigid body which implies no plastic deformation was induced during deformation. The groove and guide rolls were spinning at a speed of 0.02 rad/s. Boundary conditions of the friction on the interface between the ECAR die and billet was modeled using the constant shear model:

(9) $$\tau_{i}=m\frac{\sigma }{\sqrt{3}}$$

where $\tau_{i}\;$is the friction shear stress at the workpiece/die interface and m is the friction factor. 

The coefficient of friction between rollers and billet was set as 0.6, and the coefficient of friction between the die channel and billet was set as 0.08. The deforming body of the billet was divided into 31,150 nodes and 123,600 elements in the shape of a tetrahedron. The size of the individual elements was controlled by the size ratio (1:3) and weighting factors of strain distribution. An automatic remeshing was used in this model to prevent the heavy distortion of the mesh due to large deformation. For the FEM solution, the conjugate-gradient solver was used.

## 3. Results

### 3.1. The ECAR Processing

**Figure 3:**
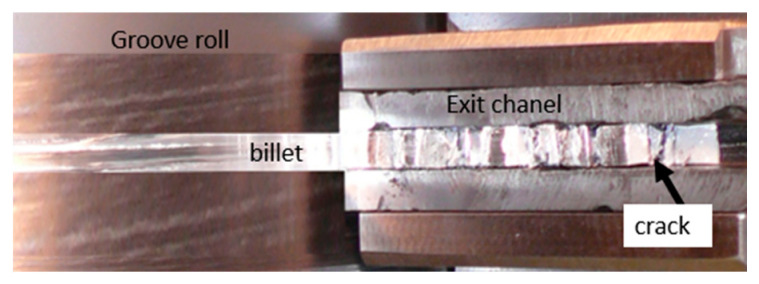
The billet of Alumix 321 after one ECAR pass.

During the ECAR processed in the first pass, the sample damage occurred in the exit channel, as shown in Figure 3 (normal direction view, without top die). Some cracks on the top surface which extend across the entire width of the billet can be seen. The sample disintegrated into segments after being removed from the channel. The results of this experiment indicated that the alloy prepared using sintered metal powder does not have sufficient formability to be processed by the ECAR process. Therefore, as part of this study, the material properties of the alloy will be investigated and will determine its limit stress values that lead to its failure. A detailed stress–strain analysis of the ECAR process using FEM simulations will also be performed, so that critical areas of the ECAR process can be identified.

### 3.2. Formability of Alumix 321

**Figure 4:**
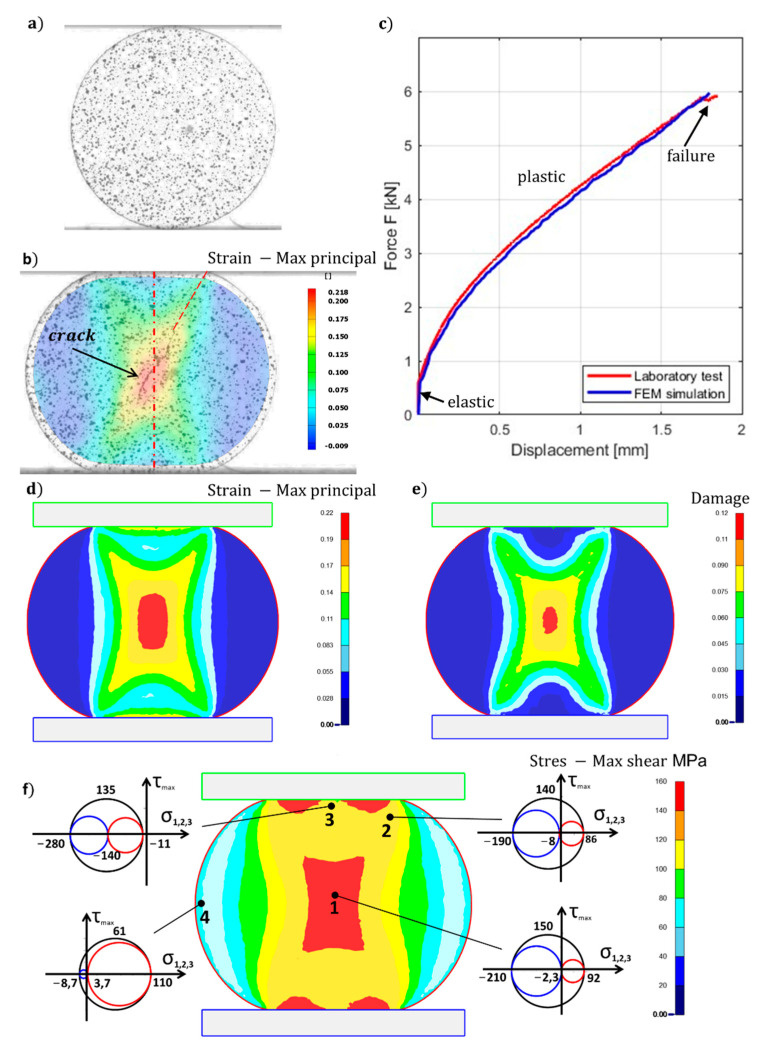
The diametrical compression test: (**a**) sample before the DC test with the pattern; (**b**) sample after the DC test with the pattern and distribution of principal strain ($\varphi_{1}$) using DIC analyses; (**c**) force comparison between FEM simulation and laboratory DC test; (**d**–**f**) distribution of the principal strain ($\varphi_{1FEM}$ ), the damage factor and max. shear stress ($\tau_{max}$ ) from FEM simulation (displayed in the plane xy), respectively.

For the critical workability values of the material Alumix 321, the DC test method was used. The deformation process of the DC test was analyzed by the DIC system [45,46,47] and subsequently compared with the FEM simulation. Figure 4a shows the pattern on the surface of the test samples before the test. Here, we can notice that at the beginning of the test the contact with the tool is very small. Figure 4b shows the state at the moment of crack formation which occurred at 18% deformation of the disc diameter in the direction of the applied force. Continuous pressing leads to an increase in the contact area of the tool which together with contact friction causes an inhomogeneous distribution of strain in the cross-section of the disk. Three deformation zones are therefore created: I is the active plastic deformation zone (Figure 4f point 1, 2), II is the cone-shaped rigid zone (Figure 4f point 3), III is the passive plastic deformation zone (Figure 4f point 3) as it is with the upsetting of the cylindrical test. The largest principal strain values ($\varphi_{1}=0.21$) are concentrated in the central part of the disk, as shown by the color scale of the strain map obtained from the DIC analysis (Figure 4b). The FEM simulation of the DC test showed the same distribution of principal strain as the DIC analysis, as shown in Figure 4d. Figure 4c presents the graphic dependence of force versus displacement, where three areas can be observed: an area of elastic deformation, a significant area of plastic deformation, and an area of failure. The failure is accompanied by a slight decrease in force. The force curve from the FEM simulations correlates with the measured values of the DC test. The distribution of maximum shear stress (Figure 4f) also clearly delimits individual deformation zones which are supplemented by Mohr’s circle of three-dimensional state for a clearer description of the state of tension of stress at the marked points. 

Point 1 represents the stress state when the cohesion of the material was broken. In this region is the material subjected to both tensile $\sigma_{1}=92\;MPa$ and compression stresses $\sigma_{2}=-2.3\;MPa$, and $\sigma_{3}=-210\;MPa$, while compressive stresses are dominant and high shear stress $\tau_{max}=150\;MPa$. Such conditions are characteristic of the initial stage of the blanking process [21]. The point 2, which also belongs to the active plastic deformation zone, has the same character as point 1, while the shear stress is slightly lower $\tau_{max}=140\;MPa$. The stress conditions at point 3 are dominated by relatively high-pressure hydrostatic stresses but also relatively high shear stress $\tau_{max}=135\;MPa$ that deforms the curved area of the disk under the anvil. The area around point 4 comprise all components of the stress tensor positive, but due to low shear stresses ($\tau_{max}=61\;MPa$) those do not disrupt the cohesion of the material. 

The damage factor of the DC test at the time of crack formation is shown in Figure 4e. A crack occurs when the damage factor reaches the value C=0.12. If the material Alumix 321 is exposed during plastic deformations to such stress–strain conditions when the damage factor is greater than 0.12, it will fail. The correctness of the measurement is also supported by the literature [48], where the same value C=0.12 was measured using the upsetting test method, which was also verified by FEM simulation.

From the analysis of the DC test of the sintered material (Alumix 321), follows that the material was broken by a ductile fracture, which was initiated in the central part of the disk due to high shear stresses and gradually spread in both directions at an angle of 30° to the direction of the force (Figure 4b). The angle of refraction essentially corresponds to the angle of the diagonal between the contact surfaces of the upper and lower pressing plates. It can be assumed that the slope of the fracture is dependent on the formability of the material. If the material properties approach the behavior of brittle materials, the slope of the fracture will decrease until it reaches the direction of the loading force as we observe when testing brittle materials such as concrete, rock, glass, etc. It should be noted here that when testing brittle materials, the fracture is always in the direction of application of the force because only normal stresses are acting at the fracture site and shear stresses are equal to zero [49].

The same fracture direction is also the case when the disk of brittle materials is loaded with flat loading platens or curved loading jaws as documented by experiments and FEM simulations [50,51,52]. Violation of the cohesion of the material occurred at the force $F_{max}=5.86\;kN$ using relation (2) getting the value of the maximum tensile stress $\sigma_{1}=96\;MPa$. This value is consistent with the results of the FEM simulation. The calculated and simulated value ($\sigma_{1}=92\;MPa$) refers to the stress conditions of the DC test and does not represent the tensile strength limit of the given material.

### 3.3. FEM Simulation of the ECAR Process

**Figure 5:**
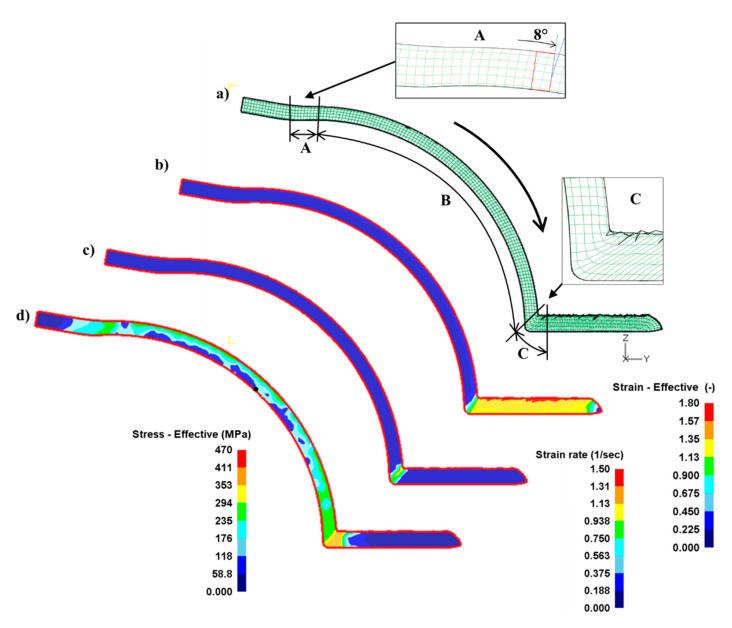
The distributions of: (**a**) the flow net; (**b**) effective strain; (**c**) strain rate effective; (**d**) stress effective in the ECAR process. The A, B, and C represent deformation zones: A—Shape Rolling, B—Upsetting, and C—SPD.

The finite element method is a numerical analysis procedure that helps in complex analyses of the deformations process and reveals critical areas of the process. The stress, strain, and strain rate distribution in the middle plane of the billet in the rolling direction during the first pass of the ECAR process is shown in Figure 5.

The flow net (Figure 5a) exhibits only two places of distortion in the grid in zone A and C, respectively. Zone A is a vertical line in the top area of the groove slightly curved (about 8°) in the direction of the filling of the groove. In the B zone, the grid does not change. The significant change of the distortion grid is in zone C, which represents simple shear in the main deformation zone. The distribution of effective strain (Figure 5b) in the exit channel achieves a high value which is characterized by a slight heterogeneity between the lower and upper edges of the billet. When compared to the ECAP process, the value of the effective strain is higher by approximately 0.6, while the distribution of heterogeneity across the billet is very similar [53]. The effective strain rate is concentrated in a narrow band at the intersection channels that define the area of simple shear (Figure 5c).

The strain rate is dependent on the speed of the billet movement through the die channel. In the case of this simulation, the speed of the billet was $4\;mm.s^{-1}$. The maximum value of strain rate $1.2\;s^{-1}$ is focused on the outer radius of the top die and slowly decreases towards the outer radius $0.8\;s^{-1}$. The distribution of effective stress is displayed in Figure 5d, where are two fields the effective stress in the groove of about 250 MPa and the effective stress in the exit channel with a maximal value of 420 MPa.

**Figure 6:**
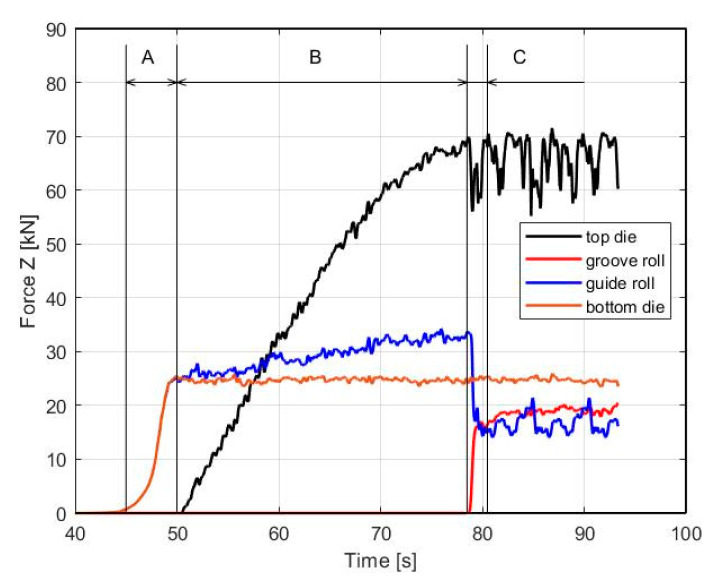
The force load in the *Z*-axis direction on the guide, grove roll, top, and bottom die during the ECAR processing. The A, B, and C represent deformation zones: A—Shape Rolling, B—Upsetting, and C—SPD.

The stresses in the billet are dependent on the contact forces acting on it from the tools. Figure 6 shows the force load in the *Z*-axis direction on the guide and grove roll and top and bottom die. The guide roll has a constant value of the rolling forces of 23 kN, whereas the rolling forces of the groove roll slowly increase up to the moment when the billet passes into the horizontal channel, then the force oscillates around 18 kN. The top die bears the greatest load because it has a large contact area with the billet. The bottom die transfers the forces related to the change in material flow from a guide roll to the exit channel.

**Figure 7:**
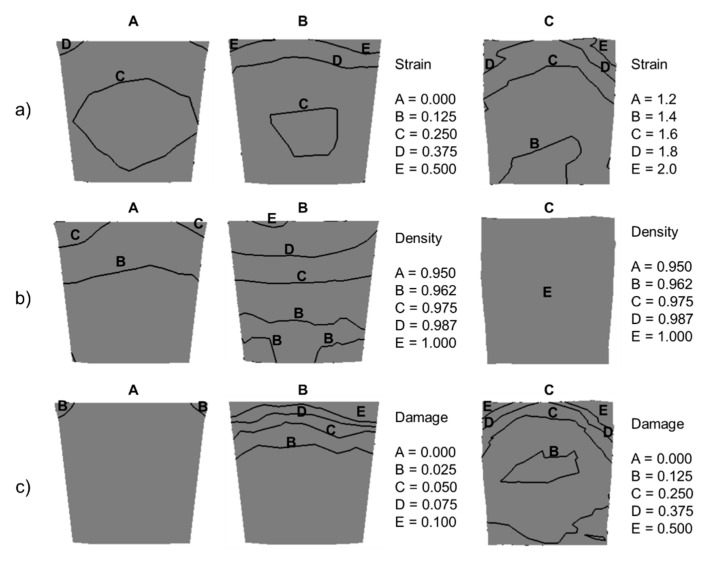
The distribution of: (**a**) effective strain; (**b**) density, and (**c**) damage factor in cross section of the billet in three zones during the ECAR process. The A, B, and C represent deformation zones: A—Shape Rolling, B—Upsetting, and C—SPD.

According to the stress–strain analysis in Figure 5 and Figure 7, the ECAR process is possibly divided into three deformation zones: Shape Rolling (zone A), Upsetting (zone B), and SPD (zone C).

Shape Rolling (zone A): it can be characterized as a primary gripping zone, where the metal fills the groove gradually by guide roll and acquires a rounded shape along its length (Figure 5). Complete canal filling can only be achieved if the groove is sufficiently overfilled with metal. The groove has a slightly trapezoidal shape for better filling and transition to the exit channel. The maximum strain is concentrated in the corners of the groove, where $\varphi_{A}=0.37,\;$as shown in Figure 7a, in the cross-section of the billet. 

**Figure 8:**
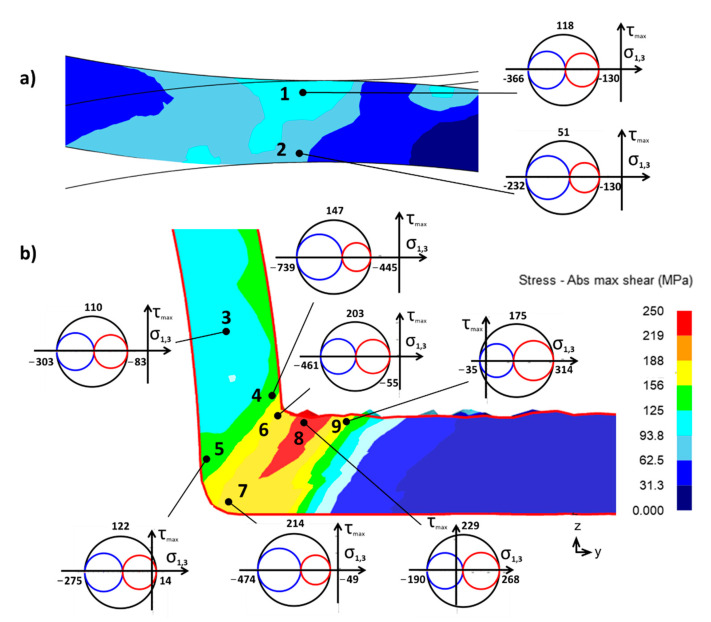
The stress state analysis of the ECAR process in: (**a**) Shape Rolling zone (points 1, 2); (**b**) part of the Upsetting zone (point 3), and SPD zone (points 4–9).

Figure 7 shows the stress state analysis in the whole ECAR process. The shape rolling section is characterized by high triaxial compressive stress as shown by Mohr’s circle in points 1 and 2 (Figure 8a). The higher maximal shear stresses ($\tau_{max}=118\;MPa$) in the upper part of the billet occur due to the creation of a trapezoidal cross-section of the billet. High triaxial compressive stress also favorably contributes to the additional compaction of porous materials. From the FEM simulation result, the density in section A (Figure 7b) was increased by about 6–7%, whereas the damage factor has a negligible value below 0.025 (Figure 7c).

Upsetting (zone B) it can be characterized as a secondary gripping zone. There is gradual increase in contact stress between the billet and groove wall from the point at which yielding occurs up to the point at which shearing deformation of the billet occur. The gradual filling of the channel also increases the contact pressure between the billet and the top die, which gradually increases to a force value of 70 kN. This force is also transmitted to the lower roll to 32 kN (Figure 6).

In this section are two areas of friction that affect the process positively but also negatively. The active–positive area creates a contact interface between the billet and groove, where high frictional forces have a favorable effect on the processing process, thanks to which the material is pushed through the main deformation zone. The size of these forces also depends on the length of the groove, that is, on the diameter of the cylinders.

A negative area of friction is created at the contact between the billet and top die, where the frictional forces act against the direction of movement of the billet, and they can also create local tensile stresses in the billet. Lubricating this contact surface is complicated in practice, so it is necessary to make the surface treatment of the tool so that the friction value does not exceed the value ranged around f=0.08.

In the entire groove, the billet is under hydrostatic pressure as shown by Mohr’s circle in point 3 in Figure 8b. The presence of high shear stresses ($\tau_{max}=110\;MPa$) promotes the compaction of porous material, where before the end of this deformation zone up to 100% density is reached in the upper part of the billet. Compaction of porous materials under the influence of high hydrostatic pressure was confirmed also during forward extrusion [54,55]. Before the end of zone B, the effective strain has been increased to value $\varphi_{B}\approx 0.37$ (Figure 7a). This change can also be caused by the compaction of the porous material (Figure 7b). In the lower part of the billet, the damage factor in this part of processing does not change (0.075) but increases to a value of 0.1 in the upper part (Figure 7c).

**SPD (zone C)**: it can be characterized as a main shearing deformation zone (MSDZ). It can be identified from the effective strain rate (Figure 5c), which from the side of the inner radius reaches a maximum value of ($\dot{\varphi_{C}}=1.2\;s^{-1}$). 

The location of the main deformation zone can also be seen in Figure 6, where the force on the knife increased sharply (red line) to 20 kN with a simultaneous decrease in the force on the lower cylinder (blue line) by 16 kN. The effective strain distribution is uneven along the SPD area (Figure 8a). The largest values are reached $\varphi_{max}\approx 1.8$ on the upper side of the billet, while it gradually decreases to the value $\varphi_{max}\approx 1.2$ towards the bottom. This heterogeneity can be eliminated by repeated ECAR processing, which contributes to further strengthening of the material.

Figure 8b shows the distribution of maximum shear stresses around the SPD zone. From the stress analysis from Mohr’s circles, it can be seen that the largest compressive stresses $(\sigma_{1,2,3}$) are around the inner radius at point 4. The severe plastic deformation zone occurs due to high shear stress which occurs under high hydrostatic pressure in the intersection of the channels as presented by Mohr’s circles in points 6 and 7.

At point 8, there is a sharp change in which tensile stress $(\sigma_{1}=268\;MPa$) dominates at a high value of shear stress $(\tau_{max}=229\;MPa$). This state of stress is likened to pure shear in plane strain conditions, which probably causes a violation of the cohesion of the material. The damage factor in this place reaches a value of up to 0.5 (Figure 8c), which is several times higher than the value measured using the DC test, where C=0.12.

Presented material violation during the ECAR processing applies only to materials with reduced formability but metals with high formability such as bulk Cu or Al do not show a violation even after multiple ECAR passes, where in the case of OFHC copper, up to 33 ECAR passes were carried out [56].

**Figure 9:**
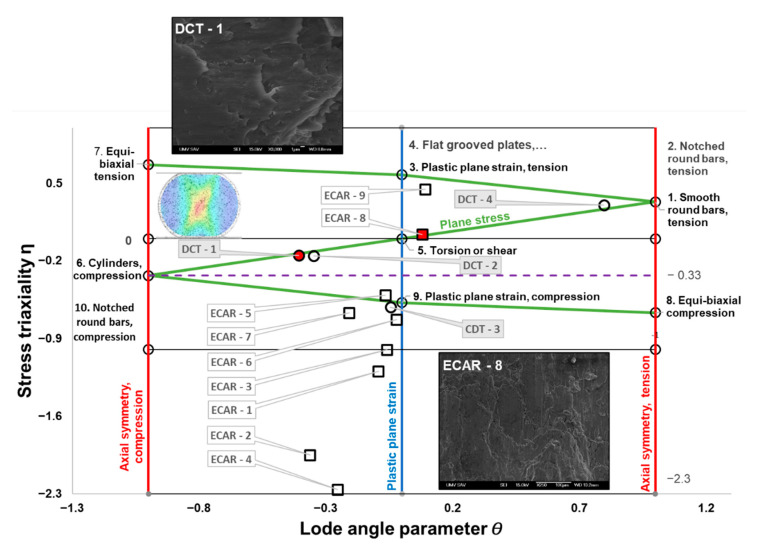
The stress state diagram shows the relationship between failure mode and stress state for the conditions of the ECAR process and the DC test.

The adverse effect of high tensile stresses (Figure 9) in the output channels, which are the cause of material failure, can be reduced or largely eliminated by changing the geometry of the output channel. The output channel can have the shape of the final product, the cross-section of which is different from the input channel. To produce such profiles, it is necessary to include in the process not only the shape matrix but also the so-called expansion chamber, in which the flow of material would be directed. Such an extrusion zone is used in COMFORM such as in the article [57], where the authors analyzed the production of the U profile using FEM simulation. The damage factor in the molding did not exceed the value of 0.11.

## 4. Discussion

When processing metals by plastic deformations, it is necessary to know the stress states that the material undergoes during processing, which is currently offered very reliably by FEM simulations. A comprehensive view of the stress state concerning limit deformations causing material failure is often evaluated based on the triaxiality stress parameter and the Lode parameter.

### 4.1. Distribution and Evolution of Stress Triaxiality

The stress triaxiality and Lode angle parameter of the DC test and the ECAR process are shown in Figure 9. The calculation of these parameters was based on Equations (6) and (8), and the principal stress $(\sigma_{1},\sigma_{2},\sigma_{3}$) and stress deviation tensor are obtained by finite element simulation. In the graphic dependence of $\eta \;versus\;\bar{\theta }$, all stresses of the ECAR process and the DC test, which were analyzed in the previous part, are shown (Figure 4 and Figure 8). The areas where the violation occurred are highlighted in red. Figure 8 also contains the stress states of the basic tests [41], which cover a wide range of stress triaxialities, which in most cases lie at the nodal points of the Z-shaped curve.

### 4.2. The Stress Triaxiality of the DC Test

During the DC test, the cohesion of the material was broken in the place marked as DCT-1 (point 1 in Figure 4). This point is located on the Z curve between the conditions of pure shear and the conditions of axial symmetry compression, this state of tension is characteristic of plane stress conditions, since$\;\sigma_{2}$ is close to zero (see Mohr’s circle in Figure 5). Shear deformation $(\bar{\theta }=-0.407$) occurs under the influence of higher compressive stresses $\left(\sigma_{3}\right)$, i.e., with negative stress triaxiality (η=−0.149). From experimental analyzes of fracture surfaces that occur in the negative stress triaxiality range (−1/3<η<0) where “shear decohesion” mode dominates [58]. In the case of the DC test, this type of failure is indicated by the fracture microstructure shown in Figure 9. 

The triaxiality stress derived in this way is caused by the geometry of the tested sample and its method of stressing. From the above analysis, we can conclude that the DC test offers an interesting area of stress that has not been tested so far. It should be noted that a similar stress area was tested with a relatively complicated geometry of butterfly shape (specifically with grove −10°) [41]. Therefore, the DC test, as a simple and relatively well-known test, can also be used as one of the tests for calibration and verification of various fracture criteria. This method still needs to be verified on other types of materials as well.

Given that the point DCT-2 lies in the area of intense deformation (see Figure 4), its stress triaxiality has the same character as point CDT-1. This state of tension will not stimulate the formation of new cracks but will support the propagation of already-formed cracks. At point CDT-4, the stress triaxiality approaches the limit values, which can trigger fracture due to high tensile stresses in the case of higher deformations on the surface of the sample.

### 4.3. The Stress Triaxiality of the ECAR Process

Material failure during the ECAR processing occurred in the exit channel at a location around point 8 (see Figure 8), which according to the triaxiality of the stress, occurred in conditions close to pure shear. At point 8, the stress triaxiality value is η=0.039 and the Lode angle value ($\bar{\theta }=0.079$) is very low. Tensile tests on samples in the form of shear in situ showed that under conditions of pure shear when $\eta ;\;\bar{\theta }\approx 0$ shear dominant fracture occurred because the voids nucleated only with a negligible growth rate and shear fracture then occurred along the slipping band [22]. The microstructure of the fracture (ECAR-8) in Figure 9 indicates that the failure occurred by shear fracture. In case the high-stress triaxiality range 0.4<η where “void nucleation, growth and coalescence” dominates. Such a failure mode may occur at point 9 but due to the low shear stresses, the fracture is not expected to occur. 

In shape rolling and using zones high negative triaxiality stress $\eta \in \;\langle -1;-1.5\rangle$, while the stress state is close to the plane strain condition. Before entering the SPD zone, there are enormous differences in negative triaxiality stress between the inner (point 4 η=−2.26) and the outer radius (point 5 η=−0.51), while in the theoretical shear plane (at the intersection of the channels) this difference is minimal (point 6 η=−0.73 and point 7 η=−0.67. Around the inner radius of the ECAR channel at points 4, 6, 8, and 9, it is possible to see how the triaxiality stress develops from extremely negative to high positives. Bao and Wierzbicki [58], an important feature of fracture was found, that a cut-off value of the stress triaxiality equal to −1/3, below which fracture never occurs. Based on these extensive analyses, it is possible to assume that in points 1–7, there will be no material failure due to the high negative stress triaxiality.

## 5. Conclusions

In this paper, the workability of sintered powder material of aluminum alloy (Alumix 321) through the ECAR process was examined. The summary of the main survey results is as follows:

- the DIC and FEM were used to study the stress state of the ECAR process and the DC test;
- the relationship between the stress state parameters and failure mode was evaluated using the stress triaxiality versus Lode parameter diagram;
- the damage factor was determined from the DC test, which reaches a value of 0.12 in the place of the crack;
- from FEM simulations is possible to divide the deformation process of the ECAR processing into three deformation areas: shape rolling zone, upsetting zones, and SPD zone, which take place under the plane strain conditions;
- due to the high-pressure hydrostatic stresses in the deformation zones, the material was probably compacted close to 100%;
- the damage factor in the output channel of the ECAR process reaches a value of up to 0.5, which is several times higher than the value measured using the DC test;
- materials with reduced formability, such as sintered PM material, cannot be additionally ennobled by plastic deformations in the ECAR process because in the main deformation zone of the process stress conditions for the pure shear are created, which will cause material violations by shearing fracture;
- when evaluating the formability of the PM material by the DC test, it was found that the DC test can be used for the calibration and verification of various fracture criteria for the stress state located between the pure shear and the axial symmetry compression.

## Data Availability

Not applicable.
